# Rib fracture after stereotactic radiotherapy for primary lung cancer: prevalence, degree of clinical symptoms, and risk factors

**DOI:** 10.1186/1471-2407-13-68

**Published:** 2013-02-07

**Authors:** Atsushi Nambu, Hiroshi Onishi, Shinichi Aoki, Licht Tominaga, Kengo Kuriyama, Masayuki Araya, Ryoh Saito, Yoshiyasu Maehata, Takafumi Komiyama, Kan Marino, Tsuyota Koshiishi, Eiichi Sawada, Tsutomu Araki

**Affiliations:** 1 Department of Radiology, University of Yamanashi, Chuo City, Yamanashi Prefecture, Japan; 2 Current institution: Department of Radiology, Teikyo University School of Medicine University Hospital, Mizonokuchi, Kawasaki City, Kanagawa Prefecture, Japan; 3 Department of Radiology, Kofu Municipal Hospital, Kofu City, Yamanashi Prefecture, Japan; 4 Department of Radiology, Yamanashi Prefectural Central Hospital, Kofu City, Yamanashi Prefecture, Japan

**Keywords:** Stereotactic body radiotherapy, Lung cancer, Rib fracture, Chest wall injury

## Abstract

**Background:**

As stereotactic body radiotherapy (SBRT) is a highly dose-dense radiotherapy, adverse events of neighboring normal tissues are a major concern. This study thus aimed to clarify the frequency and degree of clinical symptoms in patients with rib fractures after SBRT for primary lung cancer and to reveal risk factors for rib fracture. Appropriate α/β ratios for discriminating between fracture and non-fracture groups were also investigated.

**Methods:**

Between November 2001 and April 2009, 177 patients who had undergone SBRT were evaluated for clinical symptoms and underwent follow-up thin-section computed tomography (CT). The time of rib fracture appearance was also assessed. Cox proportional hazard modeling was performed to identify risk factors for rib fracture, using independent variables of age, sex, maximum tumor diameter, radiotherapeutic method and tumor-chest wall distance. Dosimetric details were analyzed for 26 patients with and 22 randomly-sampled patients without rib fracture. Biologically effective dose (BED) was calculated with a range of α/β ratios (1–10 Gy). Receiver operating characteristics analysis was used to define the most appropriate α/β ratio.

**Results:**

Rib fracture was found on follow-up thin-section CT in 41 patients. The frequency of chest wall pain in patients with rib fracture was 34.1% (14/41), and was classified as Grade 1 or 2. Significant risk factors for rib fracture were smaller tumor-chest wall distance and female sex. Area under the curve was maximal for BED at an α/β ratio of 8 Gy.

**Conclusions:**

Rib fracture is frequently seen on CT after SBRT for lung cancer. Small tumor-chest wall distance and female sex are risk factors for rib fracture. However, clinical symptoms are infrequent and generally mild. When using BED analysis, an α/β ratio of 8 Gy appears most effective for discriminating between fracture and non-fracture patients.

## Background

Stereotactic body radiotherapy (SBRT) has emerged as a new treatment for stage I lung cancer. Various articles have reported promising treatment effects [1-6]. SBRT has now come to be applied not only to medically inoperable patients, but also to operable patients. In the near future, SBRT may become feasible as an alternative to surgery for stage I non-small lung carcinoma.

Posttreatment sequelae represent an important aspect of treatment that should always be taken into account when choosing treatment options. As SBRT is an extremely dose-dense therapy, very high doses are received by normal structures adjacent to the irradiated tumor. Various unpredictable adverse events may thus arise after SBRT. Several studies have reported complications related to SBRT for lung cancer, including radiation pneumonitis [7] and chest wall injuries such as rib fracture [8-10]. The reported frequencies of rib fracture after SBRT are generally higher than those associated with other methods of radiotherapy, such as tangential breast irradiation in breast-conserving therapy. However, the reported frequencies differ widely among investigators. We speculated that such discrepancies might be largely attributable to differences in the methods used to obtain the frequencies, with authors calculating frequencies based on symptomatic patients tending to report lower frequencies than using follow-up computed tomography (CT). That is, a substantial number of patients with rib fracture appear asymptomatic. If an adverse event often proves asymptomatic, clinicians should not overemphasize the risks.

The present study therefore aimed to clarify the frequency and degree of clinical symptoms in patients with rib fracture and related chest wall injuries found on follow-up CT after SBRT. In addition, we tried to identify the threshold biologically effective dose (BED) for rib fracture after SRT and risk factors for rib fracture.

## Methods

All study protocols including chart review were approved by the institutional review board, and written informed consent was obtained from each patient for both SBRT and participation in this investigation of SBRT-related rib fracture. Clinical symptoms and imaging findings were investigated prospectively, while dosimetric details were reviewed retrospectively.

### Patients

Between November 2001 and April 2009, a total of 210 patients with primary non-small cell lung carcinoma underwent SBRT as the first treatment with a curative intent in our institution. Of these, 177 patients agreed to participate in this study. The remaining 33 patients did not participate because they were unable to visit our hospital as required in the schedule defined in the study protocol.

### Radiotherapeutic methods

SBRT was performed using noncoplanar dynamic arcs or multiple static ports. A total dose of 48–70 Gy at the isocenter was administered in 4–10 fractions at the minimum dose point in the planning target volume (PTV) using a 6-MV X-ray, comprising three different methods: 48 Gy/4 fractions; 60 Gy/10 fractions; and 70 Gy/10 fractions (Table 1). The border of the PTV was almost on the 80–85% isodose line of the global maximum dose in the PTV.

**Table 1:** Characteristics of the 177 primary lung cancer patients and the tumors

	**Lung cancer patients (n = 177)**
Mean age (range)	77.3 ± 7.0 (55–92)
Gender (male: female)	132:45
Pathology of the tumor (Ad: SCLC: SCC:spindle cell carcinoma*: unspecified**:unknown***)	89:7:47:1:9:24
Tumor diameter (average ± standard deviation)	8–55 mm(30.0 ± 9.1)
Tumor-chest wall distance (median)	0–53 mm(6)
**Range of follow-up period (median)	6–95 months (23)
Method of radiotherapy (48 Gy/4fr:60Gr/10fr:70Gr/10fr)	95:45:37
BED_10_ of the isocenter (median)	96–119 Gy (105.6)

Abbreviations: *Ad* = adenocarcinoma, *SCLC* = small cell lung cancer, *SCC* = squamous cell carcinoma, *BED* = biologically effective dose.

*Suspicious of pleomorphic carcinoma, but a definitive diagnosis was not made because of needle biopsy.

**“unspecified” indicates pathologically definitive non-small cell lung carcinoma, but unspecified for the subtype.

***“unknown” indicates clinically highly suspicious for lung cancer but no pathology obtained.

After adjusting the isocenter of the PTV to the planned position with a unit comprising a CT scanner and linear accelerator, irradiation was performed under patient-controlled breath-holding and gated radiation beam switching.

### Follow-up of patients

Every patient was basically asked to visit our clinic at 3 and 6 months after the completion of radiotherapy, and every 6 months thereafter. At every visit, a thorough examination was performed, consisting of inquiry focusing on pain at the chest wall near the irradiated tumor and respiratory symptoms, physical examination by an attending radiation oncologist, blood testing, and CT. Clinical symptoms considered related to chest wall injury after SBRT were graded according to the criteria for pain in Common Terminology Criteria for Adverse Events, *version. 3*. Chest radiologists interpreted the results of CT just after the examinations. If the patient complained of pain, analgesics were prescribed as appropriate.

### CT examination

Preradiotherapeutic and follow-up CT were performed using a 16 multidetector row scanner (Aquilion 16; Toshiba Medical Systems, Otawara, Japan). The parameters for CT were as follows: peak voltage, 120 kVp; tube rotation time, 0.5 second; slice collimation,1.0 mm (identical to reconstruction slice thickness and slice interval); and beam pitch, 0.94. Tube currents were determined by an automatic exposure control in the CT machine and the tube current showing actual range of 110–400 mA. Contrast-enhanced CT was performed in 116 patients (67.1%) after unenhanced CT.

Data were reconstructed into 5-mm sections. Thin-section CT (slice thickness, 1 mm) was also produced for the regions that included the tumor or radiation-induced opacities targeting the affected lung.

Preradiotherapeutic CT was performed within 1 month before SRT, while follow-up CT was performed at 3 and 6 months and every 6 months thereafter.

### Methods of CT evaluation

Serial follow-up CT was evaluated regarding the presence or absence of rib fractures and chest wall edema near the irradiated tumor in addition to routine radiological assessment by either of two board-certified chest radiologists at our clinic. Rib fracture was defined as a disruption of cortical continuity with malalignment. Distance between the tumor and chest wall (tumor-chest wall distance) was also measured on preradiotherapeutic CT. The time at which each finding first appeared after the completion of SBRT was reviewed later. Presence or absence of pulmonary emphysema and maximum transverse diameter of the tumor were also assessed on pre-radiotherapeutic CT.

### Evaluation of dosimetry

**Figure 1:**
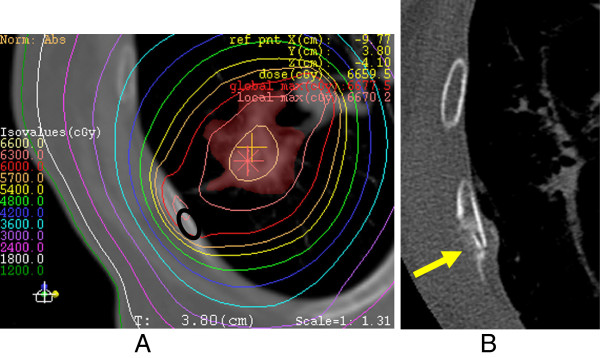
**An 86-year-old woman with adenocarcinoma after SRT. A)** Dosimetry overlaying CT with a bone window shows the maximum prescribed dose to the chest wall as 63 Gy, with a BED_3_ of 233.2 Gy. The site of rib fracture later is indicated by a black elliptical cercle. **B)** Rib fracture was noted at 24 months after completion of SRT. Amorphous osteosclerosis is also seen (arrow).

Among the 177 patients, dosimetric details were available for review in 26 patients with rib fracture and 22 patients without rib fracture (Figure 1A). Patients without fracture were randomly sampled among those with no evidence of fracture on CT for >30 months. We set this period as a cut-off point as most rib fractures after SRT in this series had occurred within 30 months after completion of SBRT. We were unable to obtain dosimetric data for the remaining patients because of breakdown of the data during the review of dosimetry. At the point that had received the maximum dose in the chest wall consisting of parietal pleura, ribs and intercostal muscles, BED was calculated in each case using a range of α/β ratios (1–10 Gy), to clarify which α/β ratio was most effective for evaluating the risk of rib fracture.

### Data analysis

First, we calculated the incidence of rib fracture after SBRT on follow-up CT in the given follow-up periods of the patients. Incidence of rib fracture was also assessed in relation to tumor-chest wall distance, and the time of rib fracture appearance was evaluated. Incidence of rib fracture was also estimated by using a Kaplan-Meier method.

Second, we calculated the frequency of clinical symptoms in patients with and without rib fractures.

Third, Cox proportional hazard model was used to identify risk factors associated with rib fracture after SBRT. The independent variables tested comprised age, sex, maximum tumor diameter, radiotherapeutic method, and tumor-chest wall distance, for which the proportionalities of hazards had been confirmed using a Kaplan-Meier method. As there were three radiotherapeutic methods, we used two dummy variables to represent them.

Fourth, receiver operating characteristic (ROC) analysis was undertaken for maximum BED of the chest wall at each α/β ratio. For BED at the α/β ratio that provided the largest area under the curve, we calculated the cut-off dose that most effectively differentiated between fracture and non-fracture patients. This was regarded as the dose at the point closest in rectilinear distance on the ROC curve to point 1.0 on the vertical axis, where both sensitivity and specificity become 1.0.

Fifth, we evaluated the correlation between the timing of rib fracture appearance and BED at the α/β ratio defined above in the 26 patients with rib fracture using Pearson’s correlation coefficient.

Values of *p* <0.05 were considered significant in all analyses. All statistical analyses were performed using IBM SPSS Statistics version 18 software (IBM, New York, USA).

## Results

### Patient demographics

Patient demographics and tumor characteristics are summarized in Table 1. Local control rates were 91% at 1 year and 83% at 3 years (detailed data not shown).

### Incidence of rib fractures after SRT

Incidence of rib fracture was 23.2% (41/177) at a median follow-up period of 33 months (range, 24–94 months) (Figure 1). When the tumor-chest wall distance was ≤25 mm, the incidence was 27.8% (41/148). The frequency of rib fracture rose to 31.3% for a distance of ≤16 mm (41/131). No patients with a distance >16 mm developed a rib fracture. When the distance was 0, the frequency of rib fracture was 36.7% (22/60). Kaplan-Meier method estimated the incidence to be 27.4% at 24 months.

### Time-to-event for rib fracture and chest wall edema

Durations to rib fracture and other related findings are summarized in Table 2. Three patients showed rib fractures ≥30 months after completion of SBRT, at 37, 53 and 58 months.

**Table 2:** Appearance times and frequencies of the rib fractures and chest wall edema

	**Appearance time ranges**	**Frequency in fracture group (n = 41)**	**Frequency in non-fracture group (n = 136)**
Rib fractures	4–58	41 (100)*	0 (0)
Chest wall edema	2–57	35 (85.4)	10 (7.4)

*The numbers in the parentheses in the frequencies of findings are percentages.

**Figure 2:**
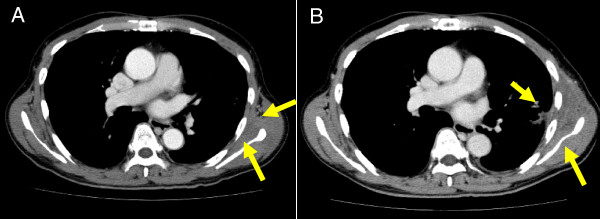
**A 64-year-old man with adenocarcinoma after SRT. A)** At 3 months after completion of SRT, contrast-enhanced CT shows suspicious findings in the left chest wall, such as slight asymmetry and indistinct intermuscular fat planes (arrows). **B)** At 15 months after SRT, contrast-enhanced CT shows definitive chest wall edema, evidenced by swelling of the left chest wall with an area of low attenuation (arrows). No rib fracture was seen at this time.

Chest wall edema was seen in 45 of 177 patients (25.4%), arising at a mean of 12 months (range, 2–57 months) (Figure 2), and appearing as asymmetrical swelling of the ipsilateral chest wall with low attenuation areas compared to the contralateral side.

### Symptoms of rib fracture

Clinical symptoms in patients with rib fracture and without rib fracture are summarized in Table 3. No patients complained of Grade 3 or more symptoms. Four patients without rib fractures complained of Grade 1 chest wall pain with all 4 cases showing radiological evidence of chest wall edema. In the study population as a whole, the frequency of chest wall pain was 21.5% (38/177). Among patients with peripheral tumors that had a tumor-chest wall distance ≤25 mm, the frequency of chest pain was 25.7%.

**Table 3:** Frequency and degree of chest wall pain

Degree of pain*	**Fracture group (n = 41)**	**Non-fracture group (n = 136)**
Grade 0	27 (65.9)**	132 (97)
Grade 1	7 (17.1)	4 (3)
Grade 2	7 (17.1)	0 (0)
Grade 3 and 4	0 (0)	0 (0)

*The degree of chest wall pain was evaluated according to Common Terminology Criteria for Adverse Events, *Ver. 3*.

**The numbers in the parentheses are percentages.

### Risk factors of rib fracture after SBRT

The results of Cox proportional hazard modeling are summarized in Table 4. Tumor-chest wall distance and sex were significant risk factors for rib fracture.

**Table 4:** Results of Cox proportional hazard model analysis

**Independent valuable**	***P*value**	**Hazard ratio**	**95% confidence interval**
Tumor-chest wall distance (mm)	<0.0001	0.858	0.801–0.920
Age	0.713	0.990	0.937–1.045
Gender*	0.002	3.172	1.503–6.695
Maximum diameter of the tumor	0.754	0.994	0.955–1.034
Radiotherapeutic method 48 Gy or not	0.829	0.911	0.391–2.122
Radiotherapeutic method 60 Gy or not	0.087	0.454	0.184–1.121

*The hazard ratio for gender is represented as the hazard of women divided by that of men.

Area under the curve ranged from 0.781 to 0.865 and was largest for an α/β ratio of 8 Gy (BED_8_). A BED_8_ value of 115.0 Gy was the most discriminative value between fracture and non-fracture patients, yielding 73% sensitivity and 91% specificity. The lowest BED_8_ that resulted in rib fracture was 91.1 Gy. BED_8_ did not correlate significantly with the timing of rib fracture appearance (r = −0.362, p = 0.070).

## Discussion

The frequency of rib fracture was 23.2% in our series. In Kaplan-Meier method, it was estimated to be 27.4% at 24 months after the SBRT. Reported frequencies of rib fracture after SBRT differ markedly among investigations, ranging from 3% to 21.2% [8-10]. Our result is closest to that described by Petterson et al., who reported the highest frequency of 21.2% by examining the results of follow-up CT [9]. We speculate that these discrepancies between studies are mainly attributable to differences in the methods of estimating frequency. Both Petterson, et al. and the present study obtained the frequency using follow-up CT, whereas other investigations have determined frequencies by confirming the presence of rib fracture on chest radiographs in patients complaining of chest pain. That is, asymptomatic patients with rib fracture may largely account for these discrepancies. In fact, only 34.1% of patients with rib fractures displayed clinical symptoms in our series. Differences in follow-up period, method of SRT and proportion of tumors close to chest wall may have also contributed to the discrepancies.

The frequency of chest wall pain was 21.5% (38/177) in our series. When confined to patients with a tumor-chest wall distance ≤25 mm, the frequency was 25.7%. Dunlap et al. observed that 17 of 60 patients (28%) with a peripheral tumor <2.5 cm from the chest wall had Grade 3 pain and another 3 patients (5%) had Grade 1 or 2 pain in the median follow-up period of 11.1 months. Frequency of chest wall pain in total was 33% in that series, higher than the present study (25.7%) despite the fact that the median follow-up period in that study was much shorter than that in the present study. Furthermore, Grade 3 pain was present in a higher proportion of the subjects in their study, compared to our investigation. We speculate that this difference is mainly due to differences in radiotherapy. BEDs at the isocenter seem much higher with the method applied by Dunlap et al.

Rib fracture is considered a common adverse event on CT after SBRT for lung cancer. However, the frequency of chest wall pain in patients with rib fracture was only 34.2%. Furthermore, pain was generally mild and usually controllable using non-opioid analgesics in our series. Reducing a prescribed dose to avoid rib fracture to such a degree the tumor may be unsatisfactorily irradiated is not justified, but using more fractions with reduced dose might help to avoid adverse events. Also, it should also be noted that the frequency and degree of chest wall pain are greatly dependent on the total dose or fractions. Our BED_10_ at the isocenter ranged from 96 to 119 Gy, which may be one of the safe ranges in terms of avoiding adverse events.

Little is known about the mechanisms by which pain is induced in chest wall injury. As most symptomatic patients had rib fracture in our series, fracture pain would be a plausible contributing factor. An animal study suggested that activation of nociceptors by mechanical stimulation or an influx of hematological and inflammatory cells into the fracture site is responsible for the pain related to fracture [11]. In contrast to rib fractures caused by trauma, those after SBRT are considered to involve a relatively chronic process and usually do not involve external force. Nociceptors might thus be less likely to be mechanically irritated, particularly in non-weight-bearing bones like ribs. In addition, radiation-induced microcapillary injury may hamper recruitment of inflammatory cells at the site of fracture in the chronic stage, which in turn may also result in less pain.

Four patients without rib fracture complained of Grade 1 pain. These patients showed evidence of chest wall edema on follow-up CT. Chest wall inflammation or contraction of the tissue due to fibrosis, which could irritate nerves, could be another factor contributing to chest wall pain.

Cox proportional hazard analysis showed that smaller tumor-chest wall distance and female sex were independent risk factors for rib fracture after SBRT while age, maximum diameter of the tumor and,radiotherapeutic method were not statistically significant risk factors. When tumor-chest wall distance decreased by 1 mm, risk of rib fracture increased by a factor of 1.153. This result is unsurprising, given that as the distance decreases, the chest wall becomes more likely to receive a high dose of radiation. In terms of sex differences, postmenopausal women are well-known to have a higher risk of osteoporosis due to decreased bone mineralization compared with men. As women in our study population were ≥55 years old, this effect most likely explains the higher likelihood of fracture in women.

ROC analysis suggested that when using BED analysis, an α/β ratio of 8 Gy was most effective for discriminating between fracture and non-fracture patients. The α/β ratios for late bone damage were estimated to be within the range of 1.8–2.8 Gy, similar to those reported for other late-responding normal tissues [12]. Our results appear to contradict those of previous reports. However, it remains unclear whether the linear-quadratic model is adaptable to hypofractionated high-dose radiotherapy, such as SBRT to the lung. In addition, we used thin-section CT, which is deemed as the most sensitive and specific examination for evaluating rib fracture. This may also partly account for the discrepancy. Our results might suggest that another model is required to estimate risks of rib fracture in SBRT.

No patients with BED_8_ <91.1 Gy developed rib fracture. Although 91.1 Gy as a maximum BED_8_ for the chest wall might suggest threshold values for rib fracture, validation studies with a larger number of patients are required.

BED_8_ did not correlate significantly with the timing of rib fracture appearance. However, we still cannot conclude that radiation dose is unrelated to the timing of rib fracture appearance, as the number of patients evaluated was too small to reach a definitive conclusion.

Several limitations to the present study must be considered. First, for BED_3_ of the chest wall, not all cases from the study population were sampled. However, we believe that our random sampling method provided a clear and concise reference value, which would offer a benchmark when considering risk of rib fracture in clinical practice. Second, although we defined the non-fracture group as cases free from rib fracture for >30 months after SBRT, a few cases developed rib fractures after 30 months. In addition, there is still a possibility of second peak in the timing of rib fracture occurrence beyond our follow-up periods. Additional cases in the non-fracture group might develop rib fractures in the future and thus should be included in the fracture group. This issue may have affected our results. Third, we estimated the time at which rib fractures and other related findings appeared as that time at which these findings were first seen on follow-up CT. However, these events would actually have occurred within the intervals from the previous follow-up CT. The present study should thus be considered to have overestimated the durations to such events. Fourth, exclusion of the 33 patients who did not participate in this study may have affected our results. However, we think that this is relatively unlikely, given that the reason for nonparticipation was that these patients were unable to visit our hospital periodically, which was considered unrelated to the frequency of adverse events.

Finally, methods of SBRT for lung cancer have yet to be standardized. In fact, our radiotherapy methods varied considerably during the period of this study. Our results therefore cannot simply be applied to patients in other institutions.

## Conclusions

Rib fracture is seen with high frequency on follow-up CT after SBRT to lung cancer. Risk factors for rib fracture after SBRT include short tumor-chest wall distance, female sex and presence of pulmonary emphysema. Clinical symptoms are infrequent and mild. When using BED analysis, an α/β ratio of 8 Gy was most effective for discriminating between fracture and non-fracture patients. However, our study was a pilot study, and thus a larger study will be required to make definitive conclusions.

## Competing interests

We have no competing interests in conducting this study and writing this paper.

## Authors’ contributions

AN did image interpretation and statistical analyses and drafted the manuscript. HO gathered the clinical data, supervised this study and edited this paper. SA gathered the clinical data and recalculated the dosimetry data. LT gathered the clinical data and investigated the relationship between radiation dose and occurrence of rib fracture. KK, MA, RS, YM, TK and KM gathered the clinical data. TK investigated the relationship between the clinical symptoms and rib fractures. ES interpreted the CT images. TA supervised this study.

## Pre-publication history

The pre-publication history for this paper can be accessed here:

http://www.biomedcentral.com/1471-2407/13/68/prepub
